# Supplementary material for: RNA structure prediction using positive and negative evolutionary information
Source: PLoS Comput Biol. 2020 Oct 30;16(10):e1008387. doi: 10.1371/journal.pcbi.1008387 (PMC7657543; doi:10.1371/journal.pcbi.1008387)
Supplement: S1 File — Includes alignments, and source code. (GZ) [file pcbi.1008387.s008.tar.gz › supplemental_material/src/rscape_v1.5.4.tar.gz!/rscape_v1.5.4/lib/infernal/infernal-1.1.2/documentation/userguide/Figures/purineC_small.pdf]

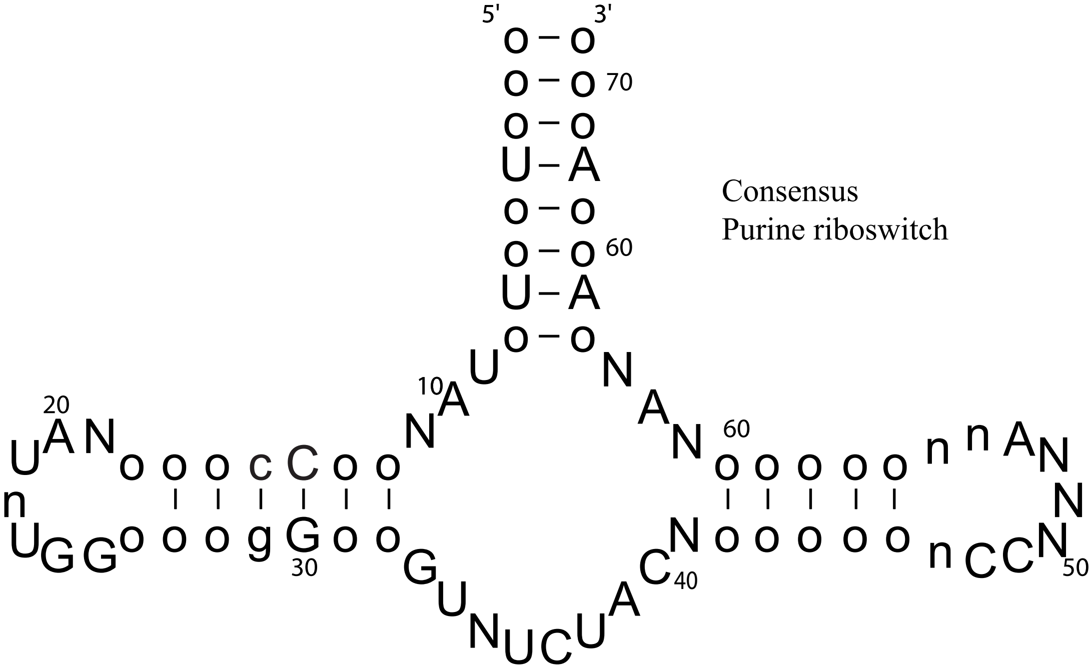


Consensus
Purine riboswitch
